# Retrospective dosimetry study of intensity-modulated radiation therapy for nasopharyngeal carcinoma: measurement-guided dose reconstruction and analysis

**DOI:** 10.1186/s13014-018-0993-2

**Published:** 2018-03-15

**Authors:** Wen-zhao Sun, Dan-dan Zhang, Ying-lin Peng, li Chen, De-hua Kang, Bin Wang, Xiao-wu Deng

**Affiliations:** State Key Laboratory of Oncology in South China, Collaborative Innovation Center for Cancer Medicine, Department of Radiation Oncology, Sun Yat-sen University Cancer Center, 651 Dongfeng Road East, Guangzhou, 510060 People’s Republic of China

**Keywords:** Intensity-modulated radiation therapy, Quality assurance, Dose reconstruction, Gamma pass rate

## Abstract

**Background:**

Conventional phantom-based planar dosimetry (2D-PBD) quality assurance (QA) using gamma pass rate (GP (%)) is inadequate to reflect clinically relevant dose error in intensity-modulated radiation therapy (IMRT), owing to a lack of information regarding patient anatomy and volumetric dose distribution. This study aimed to evaluate the dose distribution accuracy of IMRT delivery for nasopharyngeal carcinoma (NPC), which passed the 2D-PBD verification, using a measurement-guided 3D dose reconstruction (3D-MGR) method.

**Methods:**

Radiation treatment plans of 30 NPC cases and their pre-treatment 2D-PBD data were analyzed. 3D dose distribution was reconstructed on patient computed tomography (CT) images using the 3DVH software and compared to the treatment plans. Global and organ-specific dose GP (%), and dose-volume histogram (DVH) deviation of each structure was evaluated. Interdependency between GP (%) and the deviation of the volumetric dose was studied through correlation analysis.

**Results:**

The 3D-MGR achieved global GP (%) similar to conventional 2D-PBD in the same criteria. However, structure-specific GP (%) significantly decreased under stricter criteria, including the planning target volume (PTV). The average deviation of all inspected dose volumes (D_V_) and volumetric dose (V_D_) parameters ranged from − 2.93% to 1.17%, with the largest negative deviation in V100% of the PTVnx of − 15.66% and positive deviation in D1cc of the spinal cord of 6.66%. There was no significant correlation between global GP (%) of 2D-PBD or 3D-MGR and the deviation of the most volumetric dosimetry parameters (D_V_ or V_D_), when the Pearson’s coefficient value of 0.8 was used for correlation evaluation.

**Conclusion:**

Even upon passing the pre-treatment phantom based dosimetric QA, there could still be risk of dose error like under-dose in PTVnx and overdose in critical structures. Measurement-guided 3D volumetric dosimetry QA is recommended as the more clinically efficient verification for the complicated NPC IMRT.

**Electronic supplementary material:**

The online version of this article (10.1186/s13014-018-0993-2) contains supplementary material, which is available to authorized users.

## Background

Intensity-modulated radiation therapy (IMRT) is capable of improving the overall survival and long-term quality of life in patients with nasopharyngeal carcinoma [1, 2]. Patient-specific pre-treatment quality assurance (QA) is necessary for the implementation of IMRT [3], and it has been a consensus of the researcher community that patient-specific QA can be done by film dosimetry combined with ionization chambers measurement [4–6], or by a 2D/3D detector arrays test in a phantom to compare and validate the dose accuracy of the treatment [7–10]. Most of these pre-treatment QA use the ‘γ evaluation method’ for the result analysis, which is a composite analysis of distance-to-agreement (DTA) and dose difference (DD) [11–13].

The phantom measure-based γ evaluation method provides a quantitative analysis of the degree of agreement between the measured and calculated dose distributions. It can be used to confirm or evaluate if the treatment plan was delivered with sufficient accuracy based on patient-specific quality assurance. The AAPM TG-119 report [14] recommended action levels of 88% and 90% for composite and per field gamma passing rate GP (%) analysis, respectively. However, it only determines the ratio of points out of tolerance without giving any information about the spatial location of points that the dose deviated from in the origin plan, including the volumetric dose deviation for planned target volumes (PTVs) and organ at risk (OAR) of the patient [15]. Some of the recent research showed that γ passing rates of per beam planar IMRT QA did not predict clinically relevant dose errors [16], owing to a lack of correlation between the gamma passing rates (GP (%)and the volumetric dose errors in the anatomic regions-of-interest [17, 18]. It has, therefore, raised a question whether the patient OARs are safe or if the PTVs are covered by the prescribed dose when a higher passing rate is achieved.

Recently, a 3D dose reconstruction method was introduced in the IMRT QA; this method reconstructed the delivered 3D dose distribution on the patient CT image based on per beam measured doses. Olch [19] validated the software called 3DVH for 3D dose analysis of IMRT verification. In this study, the 3DVH was used to retrospectively analyze the 3D dose distribution of a group of NPC cases treated with IMRT at our center. Each treatment plan was validated with the pre-treatment 2D phantom QA and passed the 3 mm/3% GP (%) examination. The correlation between the 2D/3D GP (%) and the deviation in reconstructed DVHs were assessed as well.

## Methods

### Clinic data

The data of treatment plans for 30 NPC patients who finished IMRT treatment courses were randomized selected from our database and fully anonymized for the purpose of this retrospective analysis study. Of the total group of cases, 16 were males and 14 were females, with a sex ratio of 1.1:1. According to UICC 2009 staging criteria, there were 2, 19, 8 and 1 cases with stage II, III, IVa and IVc disease, respectively.

### IMRT Planing

All the studied cases were treated with 9-field static IMRT using a linear accelerator (Synergy, Elekta AB, Stockholm, Sweden) with 1-cm MLC and a 6 MV photon beam. The primary gross target volumes (GTV_nx_), nodal gross target volumes (GTV_nd_), and clinical target volumes (CTV_1_ and CTV_2_) were delineated manually by radiation oncologists, and the relevant planning target volumes (PTV_nx_, PTV_nd_, PTV_1_ and PTV_2_) were generated by adding a set-up margin to the corresponding volumes in all directions according to the immobilization and localization uncertainties [20, 21]. The prescribed doses were 70 Gy to PTV_nx_, 60–66 Gy to PTV_nd_, 60 Gy to PTV_1_, and 54 Gy to PTV_2_, 5 times per week with a total of 30 fractions. The dose constraints for all PTVs were that over 95% of the PTV covered by the prescribed dose, The main constrained OARs included the spinal cord, brainstem, parotid gland, temporal lobes, and larynx. All planned dose distributions were optimized and calculated with an inverse treatment planning system (TPS) (Monaco V3.0 Elekta AB, Stockholm, Sweden) using the Monte Carlo (MC) algorithm. The calculation grid was 3 mm, and 3% statistic uncertainty was used.

### Pre-treatment QA

All the 30 IMRT plans were validated with a 2D diode detector array (Mapcheck2, Sun Nuclear Corporation, Melbourne, FL). A QA plan was generated using a fractional treatment plan, and the dose distribution was recalculated in the QA phantom. The delivery of the QA plan was verified by a measurement using the diode array, and (3 mm/3%) GP (%) of greater than 90% was accepted for composite dose verification.

### Review of 3D dose reconstruction

#### 3DVH system

A commercialized 3D dose reconstruction system (3DVH, Sun Nuclear Corporation, Melbourne, FL) was used for the study, which can reconstruct 3D dose distribution in patients’ CT images based on the 2D dose distribution measured in the pre-treatment QA with a planned dose perturbation (PDP) algorithm [22]. The 3DVH software uses the dose differences between the 2D array measurement and the TPS dose calculation for each beam to produce the PDP files and then projects it back into the TPS calculated 3D dose distribution to reconstruct the delivered dose. For comparing the difference between the measurement and the original plan dose calculated by the TPS, interpolation is needed for the dose between the diode detectors of the 2D array. A so called “Smarterpolation” method is built in the 3DVH software to interpolate the measured dose to the same resolution and voxel size as the TPS calculation. The Smarterpolation estimates the dose changes in the neighborhood of every detector according to the high spatial resolution dose distribution calculated by the TPS and uses these changes to interpolate the measurement data [23, 24]. After importing the patient CT sets, RT plan, RT dose, and RT structures, the PDP files will be applied directly by the 3DVH system to perturb the planned 3D dose to produce a new 3D dose distribution in patients’ CT images, and evaluate clinically relevant dose discrepancies for each OAR or PTVs.

##### Reconstructed 3D dose analysis

Using the reconstructed 3D dose distribution, the following dosimetry related parameters were analyzed.

##### Gamma pass rate comparison

In this study, 2D GP (%) was retrieved from recorded patient QA data. A 3D dose verification review for each plan was done by the above-described PDP algorithm, and the delivered dose distributions were reconstructed on the patient CT images. The global and each organ-specific 3D GP (%) between the reconstructed dose distribution and original treatment plan were calculated using the 3DVH software. Three different criteria were used for analysis: 3 mm/3%, 2 mm/2%, and 1 mm/1%. The percentage dose differences were normalized to the global maximum dose. The GP (%) was calculated for all dose points over a threshold of 10% of the maximum dose, indicating that the detectors whose values fell within 0 to 10% would be excluded from the statistic.

##### DVH parameters comparison

To evaluate the actual delivered dose distribution and DVH deviation in patients, the reconstructed and original planned DVH parameters were compared for each of the PTVs and OAR, including: (1) dose coverage for PTVs: percentage target volume received at least 100% and 95% of the prescription dose, V_100%_ (%) and V_95%_ (%); minimum dose covered 98% and 95% of the target volume, D_98%_, and D_95%_; and mean dose in target volume, D_mean_. (2) dose for OARs: D_1cc_ of the spinal cord, brainstem, and temporal lobe (the maximum dose covering 1 cm^3^ volume of the organ); V_60Gy_ (%) of the brainstem (percentage volume that received at least 60 Gy); V_30Gy_ (%) and D_mean_ of the parotid gland (percentage volume that received at least 30 Gy dose and mean dose of the parotids); and D_mean_ of the larynx.

The percentage deviation (%) of the absolute dose and the DVH parameters were calculated using the following equations:

1 $$ \Delta D\left(\%\right)=\frac{D_{3 DVH}-{D}_{plan}}{D_{plan}}\cdot 100\% $$

2 $$ \Delta V\left(\%\right)={V}_{3 DVH}\left(\%\right)-{V}_{plan}\left(\%\right) $$

##### Correlation analysis of DVH deviation with gamma pass rate

Statistical correlation of DVH deviation (absolute value) and GP (%) was studied with the Pearson’s coefficient (r), calculated using the SPSS (19) software. The Pearson’s coefficient value of 0.8 was considered to be a significant correlation.

## Results

### Gamma pass rate comparison

For all studied cases, the GP (%) using three different criteria were evaluated for 2D, 3D, and organ-specific areas. Table 1 showed the average GP (%) of the 30 NPC cases; the maximum and minimum GP (%) values were also reported. Both the GP (%) using criteria of 3%/3 mm and 1%/1 mm for 2D planar phantom dose verification and the global 3D reconstructed dose verification were significantly different, based on the paired samples T test. Compared to the global 3D GP (%), the mean GP (%) was relatively lower in PTVs but relatively higher in the main OAR for the 3 mm/3% criterion. However, the GP (%) decreased a lot in both PTVs and some OAR when a stricter criterion (1 mm/1%) was used.

**Table 1:** The comparison of the 2D, 3D globe, and organ-specific GP (%) for 30 NPC cases with different gamma criteria

	3%/3 mm	2%/2 mm	1%/1 mm
	GP (%)	GP (%)	GP (%)
Global GP (%)			
2D phantom verification	96.4 ± 2.9 [90.1–99.7]	90.2 ± 5.5 [74.9–99.0]	60.9 ± 9.6 [41.2–84.6]
3Dreconstruction verification	97.7 ± 3.2 [86.3–99.6]	91.3 ± 6.2 [71.1–98.7]	67.8 ± 9.9 [40.6–88.7]
*p*	0.002	0.152	0.000
Organ GP (%) of 3D reconstruction verification			
PTVnx (70 Gy)	93.78 ± 11.03 [57–100]	79.24 ± 22.1 [23.1–99.8]	46.55 ± 24.83 [4–85.8]
PTV1 (60 Gy)	95.61 ± 7.31 [72.6–100]	83.12 ± 17.19 [41.2–99.9]	50.47 ± 21.37 [8–87.9]
PTV2 (54 Gy)	94.41 ± 7.48 [67.4–99.8]	81.32 ± 11.81 [55.3–99.4]	50.45 ± 15.46 [22.4–87.4]
Spinal cord	98.24 ± 2.32 [90.2–100]	89.09 ± 8.45[64.3–100]	57.43 ± 14.18[30.9–85.3]
Brainstem	99.17 ± 1.48 [93.7–100]	94.00 ± 7.41[71.9–100]	65.41 ± 19.49 [14.3–91]
Left parotid gland	99.22 ± 2.78 [84.8–100]	95.85 ± 6.01[72.3–100]	78.84 ± 12.96 [49.1–96.8]
Right parotid gland	99.25 ± 2.21 [89.9–100]	95.44 ± 6.15[75.8–100]	76.50 ± 15.47 [43.1–97.2]
Left temporal lobe	99.40 ± 1.78 [92.4–100]	95.14 ± 7.51[65.6–100]	70.03 ± 24.06 [18.2–95.1]
Right temporal lobe	99.35 ± 1.61 [92.6–100]	95 ± 6.60 [71.6–100]	69.1 ± 21.43 [11.6–96.1]
Larynx	96.52 ± 5.21 [78.4–100]	86.38 ± 13.85[50.6–100]	55.88 ± 26.23 [12.8–90.9]

*p* indicates significance on the two-tailed Student t-test

*Abbreviations*:*GP* (%)-gamma pass rate (%); *PTV* - planning target volume; *NPC* - nasopharyngeal carcinoma;

### Reconstructed DVH

**Fig. 1:**
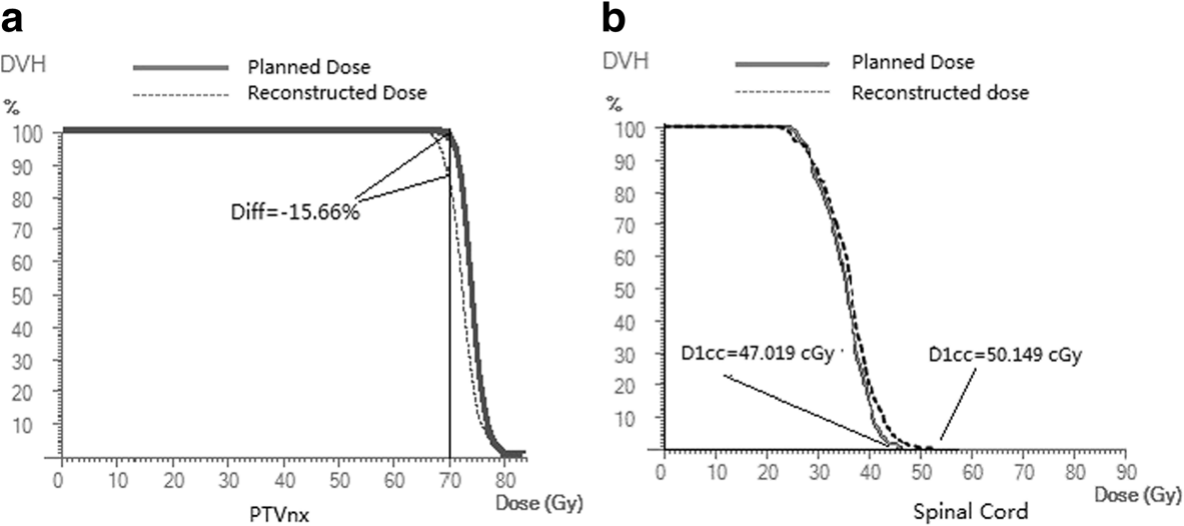
Examples of two cases with clinically significant dose deviation, (**a**) Underdose of − 15.66% in V100% of the PTVnx, (**b**) Increase of 6.66% in D1cc of the spinal cord

The average relative difference in the volumetric dose (D_V_) and dose volume (V_D_) between the 3D dose reconstruction and the planned dose ranged from − 2.93% to 0.02% for PTVs, and − 1.66% to 1.17% for OAR (Table 2). Although the average deviations were slight, clinically significant deviation was found in some individual cases. In Table 3, eight of the 30 cases were under-dosed with a discrepancy of − 5% in V70 Gy (V100%) of the PTVnx. One of the 30 cases received a 5% higher dose than the planned dose separately in D1cc of the spinal cord and the mean dose of the larynx. Fig 1 shows the two cases with the highest dose deviation in PTV and OAR, one with a largest negative deviation (− 15.66%) in V100% of the PTVnx and another case with a significant positive deviation (6.66%) in D1cc of the spinal cord.

**Table 2:** Relative deviations in D_V_ and V_D_ in PTVs and main OARs between the 3D reconstructed dose and planned dose

	V100%	V95%	V60 Gy	V30 Gy	D98%	D95%	D1cc	Dmean
PTVnx								
Δ(%)	2.93 ± 5.03	−0.84 ± 1.59			−1 ± 1.39	−0.67 ± 1.39		0.02 ± 1.52
Range (%)	[−15.66,5.25]	[−6.25,1.23]			[−3.82,1.34]	[−3.42,1.83]		[−2.93,2.97]
*p*	0.003	0.142			0.007	0.013		0.933
PTV1								
Δ(%)	−0.37 ± .062	− 0.08 ± 0.16			−1 ± 1.43	− 0.95 ± 1.32		
Range (%)	[−3.00,0.41]	[−0.63,0.04]			[−4.58,0.78]	[−3.69,1.09]		
*p*	0.003	0.016			0.00	0.00		
PTV2								
Δ(%)	−0.37 ± 0.48	− 0.14 ± 0.20			− 0.95 ± 1.43	− 0.19 ± 1.21		
Range (%)	[−1.26,1.10]	[−0.78,0.32]			[−4.15,2.47]	[−2.96,3.19]		
*p*	0.000	0.001			0.001	0.39		
Spinal Cord								
Δ(%)							0.62 ± 1.91	
Range (%)							[−3.22,6.66]	
*p*							0.088	
Brain Stem								
Δ(%)			−0.62 ± 1.2				−0.73 ± 1.84	
Range (%)			[−3.49,0.34]				[−4.80,3.84]	
*p*			0.008				0.039	
Left Parotid Gland								
Δ(%)				−1.40 ± 2.59				−1.33 ± 1.59
Range (%)				[−12.11,1.43]				[−4.69,2.00]
*p*				0.006				0.00
Right Parotid Gland								
Δ(%)				−1.27 ± 1.49				−1.45 ± 1.54
Range (%)				[−4.78,2.16]				[−4.30,1.85]
*p*				0.000				0.000
Left Temporal Lobe								
Δ(%)							−1.40 ± 2.42	
Range (%)							[−9.42,3.90]	
*p*							0.004	
Right Temporal Lobe								
Δ(%)							−1.66 ± 3.49	
Range (%)							[−16.02,3.64]	
*p*							0.014	
Larynx								
Δ(%)								1.17 ± 1.58
Range (%)								[−2.43,5.08]
*p*								0.000

Note. Δ(%) represents the relative deviation between the 3D reconstructed and planned doses (average ± standard deviation). p indicates significance on the two-tailed Student t-test

*Abbreviations*:*D*_*V*_- volumetric dose and *V*_*D*_ - dose volumes; *PTV* - planning target volume; *OAR* – organ at risk

**Table 3:** Percentage of cases with clinically significant dose deviation (more than 5% decrease in the prescribed dose coverage of PTVs or increase in the planned dose of OAR)

Parameters	PTVx(V70 Gy)	PTV1(V60 Gy)	PTV2(V54 Gy)	Spinal Cord(D1cc)	Larynx(D_mean_)
	8/30	0/30	0/30	1/30	1/30

*Abbreviations*: *PTV* - planning target volume, *OAR* - organ at risk

### Correlation analysis of DVH deviation and gamma pass rate

The results of statistical correlations between D_V_, V_D,_ and GP (%), described by Pearson’s coefficient (r), are shown in Table 4. No obvious correlations (both criteria *R* > 0.8 and *p* < 0.05 were met) were found between all the DVH metrics and the global GP (%) got from the 2D QA measurement and the 3D reconstructed dose. In the measurement-based 3D dose verification, only the reconstructed D2% and the D_mean_ of the PTV_nx_ showed a significant (*p* < 0.01) strong correlation with the organ-specific GP (%) for the PTV_nx_, when a Pearson’s coefficient value of 0.8 was used for the correlation evaluation. The plots of the correlation analysis with the R^2^ value is available in the additional figures files [see Additional file 1: Figures S1 to S10].

**Table 4:** Pearson correlation coefficient with three type gamma pass rate and D_V_, V_D_

Structure	DVH index	Planar 2D GP(%)			Global 3D GP(%)			Organ specific 3D GP(%)		
		3 mm/3%	2 mm/2%	1 mm/1%	3 mm/3%	2 mm/2%	1 mm/1%	3 mm/3%	2 mm/2%	1 mm/1%
PTVnx	V100%	−0.038	− 0.106	− 0.252	0.044	− 0.145	− 0.238	− 0.328	− 0.405	− 0.267
	D98%	− 0.143	− 0.099	− 0.217	0.010	− 0.244	− 0.391	− 0.358	− 0.425	− 0.291
	D2%	−0.336	− 0.332	− 0.460	− 0.336	− 0.398	−0.409	− 0.754	**− 0.810** ^*^	**− 0.811** ^*^
	Dmean	− 0.386	− 0.409	− 0.585	− 0.248	− 0.442	− 0.567	**− 0.836** ^*^	**− 0.968** ^*^	**− 0.935** ^*^
PTV1	V100%	−0.035	− 0.217	− 0.210	− 0.174	− 0.232	− 0.258	− 0.166	−0.189	0.056
	D95%	0.131	0.065	−0.110	0.162	−0.124	− 0.362	− 0.135	− 0.409	− 0.431
PTV2	V100%	0.046	0.152	0.118	−0.057	− 0.164	− 0.151	− 0.072	− 0.102	−0.006
	D95%	−0.168	−0.253	− 0.225	− 0.563	− 0.58	− 0.467	− 0.525	−0.458	− 0.376
SC	D1cc	−0.185	−0.157	− 0.216	− 0.309	− 0.478	− 0.443	− 0.595	− 0.664	− 0.650
BS	V60 Gy	− 0.147	− 0.260	− 0.118	− 0.111	− 0.376	− 0.421	− 0.140	− 0.384	− 0.474
Parotid-L	Dmean	0.160	0.079	0.004	0.118	0.042	− 0.050	− 0.007	− 0.173	− 0.385
	V30 Gy	0.297	0.096	− 0.115	0.158	0.017	−0.122	0.065	−0.377	− 0.403
Parotid-R	Dmean	−0.056	− 0.022	− 0.049	0.091	− 0.034	− 0.151	0.007	− 0.126	− 0.489
	V30 Gy	− 0.064	0.056	− 0.057	0.093	− 0.085	− 0.207	− 0.057	− 0.147	−0.42
TL-L	D1cc	0.043	0.146	0.139	0.151	0.132	0.087	0.068	−0.096	−0.271
TL-R	D1cc	0.055	0.162	0.196	0.058	0.035	0.050	0.370	−0.164	−0.241
Larynx	Dmean	−0.225	−0.288	− 0.243	−0.315	− 0.366	−0.363	0.079	−0.349	− 0.666

The two-tailed Student t-test with SPSS statistical software (V19);

Bold letters in the data highlighted *R* > 0.8 and ^*^ symbolled the significance of the correlation at the 0.01 level (*P* < 0.01, bilateral);

*Abbreviations: D*_*V*_ volumetric dose, *V*_*D*_ dose volumes, *PTV* planning target volume, *GP (%)* gamma pass rate (%), *SC* spinal cord, *BS* brainstem, *Parotid-L* left parotid gland, *Parotid-R* right parotid gland, *TL-L* left temporal lobe, *TL-R* right temporal lobe

## Discussions

Phantom measurement and global GP (%) evaluation are widely accepted in the radiation therapy (RT) community as a routine IMRT QA procedure. According to the report of AAPM TG119, the 3 mm /3% criterion is suggested for this kind of verification. In this study, an average GP (%) of 96.4%, ranging from 89.1% to 99.7%, was achieved using the AAPM suggested criteria. However, the GP (%) significantly decreased using a stricter acceptance criterion, which is similar to the report of Benjamin E, et al. [16], although it did not reflect a volumetric dose deviation in the PTVs and OAR.

The results of the correlation analysis showed that all the coefficient values (r) were much lower than 0.8 for correlations between the global GP (%) and D_V_ or V_D_ for each of the PTVs and OAR. It indicated either no correlation or only very weak correlation existing between the global GP (%) and the deviation of DVH parameters. M. Stasi, et al. (17) have reported similar results in their study of 2 groups of IMRT cases (prostate and pelvic IMRT, and head and neck IMRT), wherein all coefficient values were smaller than 0.8, indicating a weak correlation between the GP (%) and the dose deviation.

In the organ-specific GP (%) analysis, the GP (%) of three different criteria all showed strong negative correlation with the deviation of mean dose in the PTV_nx_-specific evaluation. A coefficient value larger than 0.8, indicated that the higher the GP (%) in the PTVnx, the less the deviation in the mean dose of its volume. Also, the strength of the correlation coefficients (r) of the organ-specific GP (%) was higher than that of the global GP (%). These results are consistent with the findings of M. Cozzlino et al [18]. In their study of a group of RapidArc treatment plans for the prostate, on using the COMPASS system (IBA Dosimetry, Germany) to reconstruct the delivered dose distribution, a stronger correlation was observed between the organ-specific GP (%) and dose deviation rather than with the global GP (%).

**Fig. 2:**
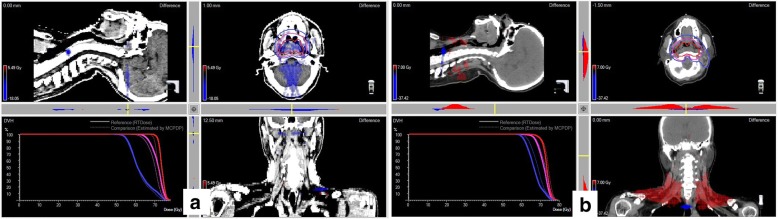
Examples of two reviewed cases. The left one (**a**) had a high global GP (%) of 99.2% with a low dose region inside the target area (in blue color) yielded a poor PTV_nx_-specific GP (%) of 85.7%. The right one (**b**) had lower global GP (%) of 90.4% but 100% PTV_nx_-specific GP (%) and an increasing dose region in the lower neck (in red color). The gamma pass rate for all the cases was calculated using the 3 mm/3% criterion

A high global GP (%) did not always mean a high organ-specific GP (%) (e.g. target volume specific GP (%)), and vice versa, a low global GP (%) did not always indicate a low GP (%) in the specific organ volumes. As depicted in Fig. 2, the case on the left one showed a high global GP (%) which meet the QA criteria, but not ensured the clinical concerned dose errors within tolerance. In fact, a significant low-dose area was located in the PTV_nx_ leading to a large reduction (12.8%) in the V_70Gy_, which might reduce local control of the treatment. The case on the right showed a relatively low global GP (%), but the dose error all distributed out of the gross tumor, high risk and critic structure areas.

M. Stasi et al. [17] observed that the measurement-based reconstructed delivery doses to the PTVs were all negative discrepant in their analysis of a group of cases of prostate and head and neck cancers using the same 3DVH system. M. Cozzolino et al [18] reported the discrepancy between the measurement-guided dose reconstruction using a 3D QA system (COMPASS, IBA Dosimetry, Schwarzenbruck, Germany) and the original plan, in which the actual dose could be 5% greater than the planned value in some cases. In our review study, the deviation of the reconstructed DVH from the planned values ranged between 6.66% and − 15.66%. There were 27% (8/30) of cases in which coverage of the prescribed dosage in the gross tumor volume (V70 Gy of the PTVnx) decreased by 5% or more, implicating the possibility of a potential effect on local control of the treatment, which was concealed during the pretreatment 2D phantom verification. In addition, there were two cases with > 5% dose increment in critical structures, separately in the D1cc of the spinal cord and in the mean dose of the larynx, compared to the planned doses. In the case of the largest dose increase, in the spinal cord, the planned D1cc was 47.019 Gy, and the reconstructed D1cc was 50.149 Gy which was already beyond our clinically tolerated dose. This big discrepancy in the dose should be noticed before treatment and carefully re-evaluated, especially in cases where the planned dose was close to the tolerated dose. Except for the above-mentioned cases, all other OARs showed a very small deviation in DVHs. A carefully review of DVHs of the PTVs and OARs revealed that these kinds of dose deviations could be overlooked when only global GP (%) evaluation is used in the pretreatment QA. Hence, a volumetric dose verification and evaluation might be needed in clinical practice by means of 3D dose reconstruction based on delivery measurement.

In this report of our study, the gamma pass rate (GP (%)) evaluation was based on the percentage dose differences normalized to the global maximum dose. This is good for the high-dose regions close to the target. However, for some organs at risk which are found in the lower-dose region, this normalization might underestimate the real difference in dose, and a local dose difference might be helpful for understanding the sensitivity of the GP (%) in some cases. For this reason, we also analyzed the GP (%) using local dose normalization and found that it was lower than that using global maximum dose normalization. Nevertheless, both GP (%) of global maximum dose normalization and local dose normalization had the similar results in the DVH correlation analysis, having no significant strong correlation with the DVH errors, except in the PTV_nx_-specific GP% and the DVH error (detail data is available in an additional table file [see Additional file 2: Table S1-S3]).

Although the measurement-guided 3D dose reconstruction method can be used to predict the actually delivery dose distribution on patient before IMRT treatment, the actual delivered dose distribution in patient, during the whole treatment course underwent a long period of time, may be affected by many factors such as the change in multi-leaf collimator (MLC) position accuracy, beam energy fluctuation, gross machine monitor (MU) errors, tumor shrink and anatomy changes, etc. The accumulated actual delivered dose distribution on patient will be interesting in our future work.

## Conclusions

Traditional 2D Phantom QA and global GP (%) evaluation is not sufficient for ensuring the clinically accurate volumetric dose for IMRT treatment, as there is no strong correlation between the global GP (%) and percentage deviation in DVH of both PTVs and OAR, even when a strict 1%/1 mm gamma criterion was used. According to the results of our study, 3D dose verification and organ-specific GP (%) evaluation is a more effective QA method, and the PTVnx specific GP (%) has a strong negative correlation with the mean dose of the PTVnx. Although the IMRT treatment plan passed a 2D phantom-based dosimetry QA of GP (%) evaluation, there is still a potential risk of volumetric dose deviation, such as lack of dose coverage in the target or an overdose in the OAR. Three-dimensional dose reconstruction based on measurement and DVH verification are recommended for IMRT QA, rather than taking the GP (%) evaluation only.

## Additional files

Additional file 1: **Figure S1.** Dose deviation (DD(%)) in PTVnx vs GP (%)-linear fits and R2 were reported. **Figure S2.** Dose deviation (DD(%)) in PTV1 vs GP (%)-linear fits and R2 were reported. **Figure S3.** Dose deviation (DD(%)) in PTV2 vs GP (%)-linear fits and R2 were reported. **Figure S4.** Dose deviation (DD(%)) in spinal cord vs GP (%)-linear fits and R2 were reported. **Figure S5.** Dose deviation (DD(%)) in Brain stem vs GP (%)-linear fits and R2 were reported. **Figure S6.** Dose deviation (DD(%)) in left Parotid gland vs GP (%)-linear fits and R2 were reported. **Figure S7.** Dose deviation (DD(%)) in right Parotid gland vs GP (%)-linear fits and R2 were reported. **Figure S8.** Dose deviation (DD(%)) in left Temporal lobe vs GP (%)-linear fits and R2 were reported. **Figure S9.** Dose deviation (DD(%)) in right Temporal lobe vs GP (%)-linear fits and R2 were reported. **Figure S10.** Dose deviation (DD(%)) in Larynx vs GP (%)-linear fits and R2 were reported. (ZIP 17676 kb)

Additional file 2: **Table S1.** The comparison of the 3D globe, and organ-specific GP (%) calculated with local dose normalization for 30 NPC cases with different gamma criteria. **Table S2.** Pearson correlation coefficient with three type gamma pass rate calculated with local dose normalization and D_V_, V_D_. **Table S3.** Significant *p*-values for correlation between three type gamma pass rate and D_V_, V_D_. (ZIP 55 kb)

## Abbreviations

2D-PBD: Conventional phantom-based planar dosimetry
3D-MGR: Measurement-guided 3D dose reconstruction
CT: Computed tomography
DD: Dose difference
DTA: Distance-to-agreement
D_V_: Volumetric dose and
DVH: Dose-volume histogram
GP (%): Gamma pass rate (%)
NPC: Nasopharyngeal carcinoma
OAR: Organ at risk
PTV: Planning target volume
QA: Quality assurance
V_D_: `Dose volumes
