# Correction to: Retrospective dosimetry study of intensity-modulated radiation therapy for nasopharyngeal carcinoma: measurement-guided dose reconstruction and analysis

**DOI:** 10.1186/s13014-018-1058-2

**Published:** 2018-06-25

**Authors:** Wen-zhao Sun, Dan-dan Zhang, Ying-lin Peng, Li Chen, De-hua Kang, Bin Wang, Xiao-wu Deng

**Affiliations:** State Key Laboratory of Oncology in South China, Collaborative Innovation Center for Cancer Medicine, Department of Radiation Oncology, Sun Yat-sen University Cancer Center, 651 Dongfeng Road East, Guangzhou, 510060 People’s Republic of China

## Correction

The original version of this article [1] unfortunately contained a mistake. The presentation of Table 4 was incorrect. The corrected table is given below and the original article was corrected.

**Table 4:** Pearson correlation coefficient with three type gamma pass rate and D_V_, V_D_

Structure	DVH index	Planar 2D GP(%)			Global 3D GP(%)			Organ specific 3D GP(%)		
		3 mm/3%	2 mm/2%	1 mm/1%	3 mm/3%	2 mm/2%	1 mm/1%	3 mm/3%	2 mm/2%	1 mm/1%
PTVnx	V100%	−0.038	− 0.106	− 0.252	0.044	− 0.145	− 0.238	− 0.328	− 0.405	− 0.267
	D98%	− 0.143	− 0.099	− 0.217	0.010	− 0.244	− 0.391	− 0.358	− 0.425	− 0.291
	D2%	−0.336	− 0.332	− 0.460	− 0.336	− 0.398	−0.409	− 0.754	**− 0.810** ^*^	**− 0.811** ^*^
	Dmean	− 0.386	− 0.409	− 0.585	− 0.248	− 0.442	− 0.567	**− 0.836** ^*^	**− 0.968** ^*^	**− 0.935** ^*^
PTV1	V100%	−0.035	− 0.217	− 0.210	− 0.174	− 0.232	− 0.258	− 0.166	−0.189	0.056
	D95%	0.131	0.065	−0.110	0.162	−0.124	− 0.362	− 0.135	− 0.409	− 0.431
PTV2	V100%	0.046	0.152	0.118	−0.057	− 0.164	− 0.151	− 0.072	− 0.102	−0.006
	D95%	−0.168	−0.253	− 0.225	− 0.563	− 0.58	− 0.467	− 0.525	−0.458	− 0.376
SC	D1cc	−0.185	−0.157	− 0.216	− 0.309	− 0.478	− 0.443	− 0.595	− 0.664	− 0.650
BS	V60 Gy	− 0.147	− 0.260	− 0.118	− 0.111	− 0.376	− 0.421	− 0.140	− 0.384	− 0.474
Parotid-L	Dmean	0.160	0.079	0.004	0.118	0.042	− 0.050	− 0.007	− 0.173	− 0.385
	V30 Gy	0.297	0.096	− 0.115	0.158	0.017	−0.122	0.065	−0.377	− 0.403
Parotid-R	Dmean	−0.056	− 0.022	− 0.049	0.091	− 0.034	− 0.151	0.007	− 0.126	− 0.489
	V30 Gy	− 0.064	0.056	− 0.057	0.093	− 0.085	− 0.207	− 0.057	− 0.147	−0.42
TL-L	D1cc	0.043	0.146	0.139	0.151	0.132	0.087	0.068	−0.096	−0.271
TL-R	D1cc	0.055	0.162	0.196	0.058	0.035	0.050	0.370	−0.164	−0.241
Larynx	Dmean	−0.225	−0.288	− 0.243	−0.315	− 0.366	−0.363	0.079	−0.349	− 0.666

The two-tailed Student t-test with SPSS statistical software (V19);

Bold letters in the data highlighted *R* > 0.8 and ^*^ symbolled the significance of the correlation at the 0.01 level (*P* < 0.01, bilateral);

*Abbreviations: D*_*V*_ volumetric dose, *V*_*D*_ dose volumes, *PTV* planning target volume, *GP (%)* gamma pass rate (%), *SC* spinal cord, *BS* brainstem, *Parotid-L* left parotid gland, *Parotid-R* right parotid gland, *TL-L* left temporal lobe, *TL-R* right temporal lobe
