# Supplementary figures and images for: Retrospective dosimetry study of intensity-modulated radiation therapy for nasopharyngeal carcinoma: measurement-guided dose reconstruction and analysis
Source: Radiat Oncol. 2018 Mar 15;13:42. doi: 10.1186/s13014-018-0993-2 (PMC5856312; doi:10.1186/s13014-018-0993-2)

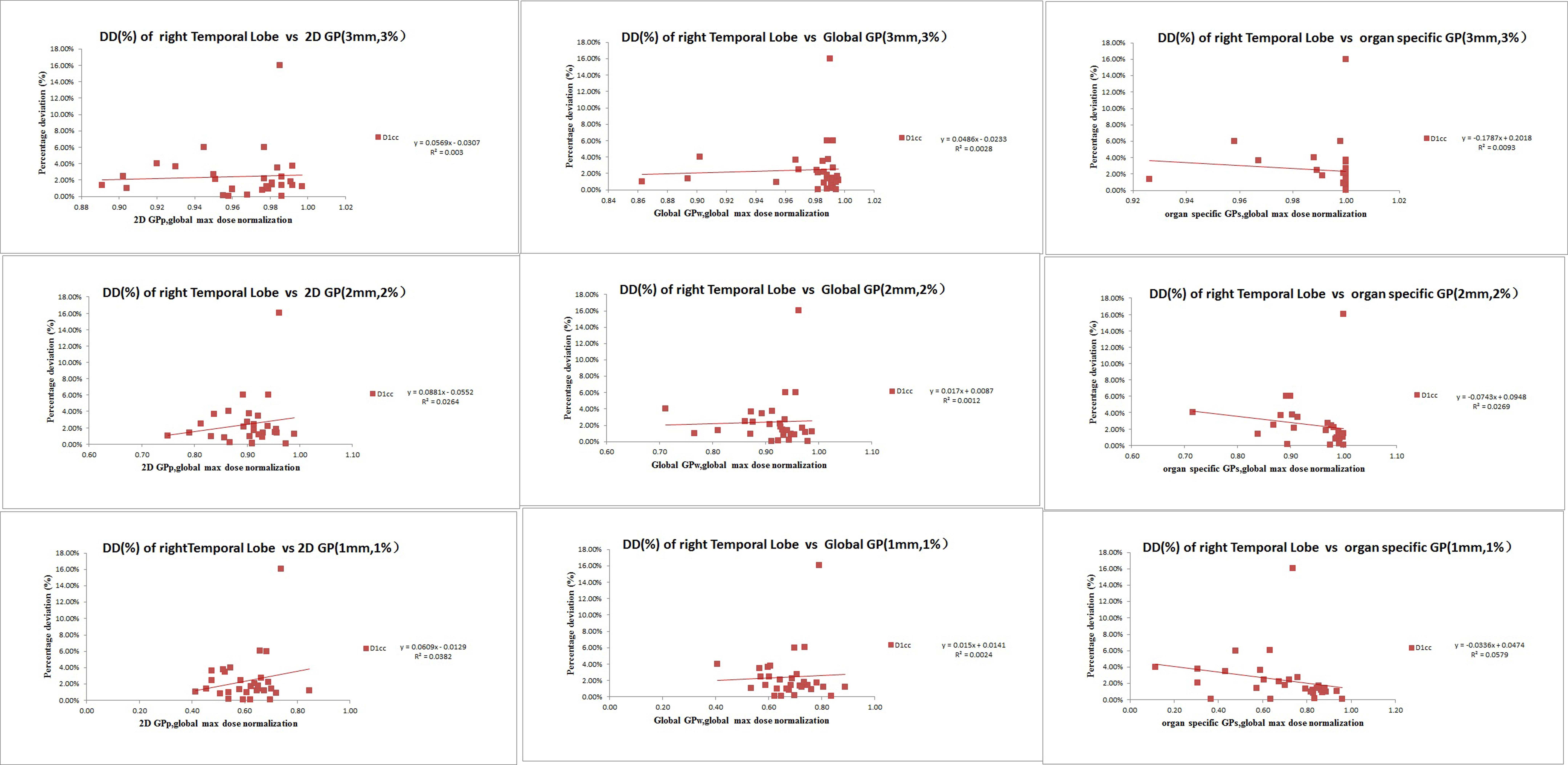

Supplement: Supplementary file 1 — Figure S1. Dose deviation (DD(%)) in PTVnx vs GP (%)-linear fits and R2 were reported. Figure S2. Dose deviation (DD(%)) in PTV1 vs GP (%)-linear fits and R2 were reported. Figure S3. Dose deviation (DD(%)) in PTV2 vs GP (%)-linear fits and R2 were reported. Figure S4. Dose deviation (DD(%)) in spinal cord vs GP (%)-linear fits and R2 were reported. Figure S5. Dose deviation (DD(%)) in Brain stem vs GP (%)-linear fits and R2 were reported. Figure S6. Dose deviation (DD(%)) in left Parotid gland vs GP (%)-linear fits and R2 were reported. Figure S7. Dose deviation (DD(%)) in right Parotid gland vs GP (%)-linear fits and R2 were reported. Figure S8. Dose deviation (DD(%)) in left Temporal lobe vs GP (%)-linear fits and R2 were reported. Figure S9. Dose deviation (DD(%)) in right Temporal lobe vs GP (%)-linear fits and R2 were reported. Figure S10. Dose deviation (DD(%)) in Larynx vs GP (%)-linear fits and R2 were reported. (ZIP 17676 kb) [file 13014_2018_993_MOESM1_ESM.zip › Additional figure-9. Dose deviation in right Temporal lobe vs GP (%)-linear fits and R2 were reported..jpg]

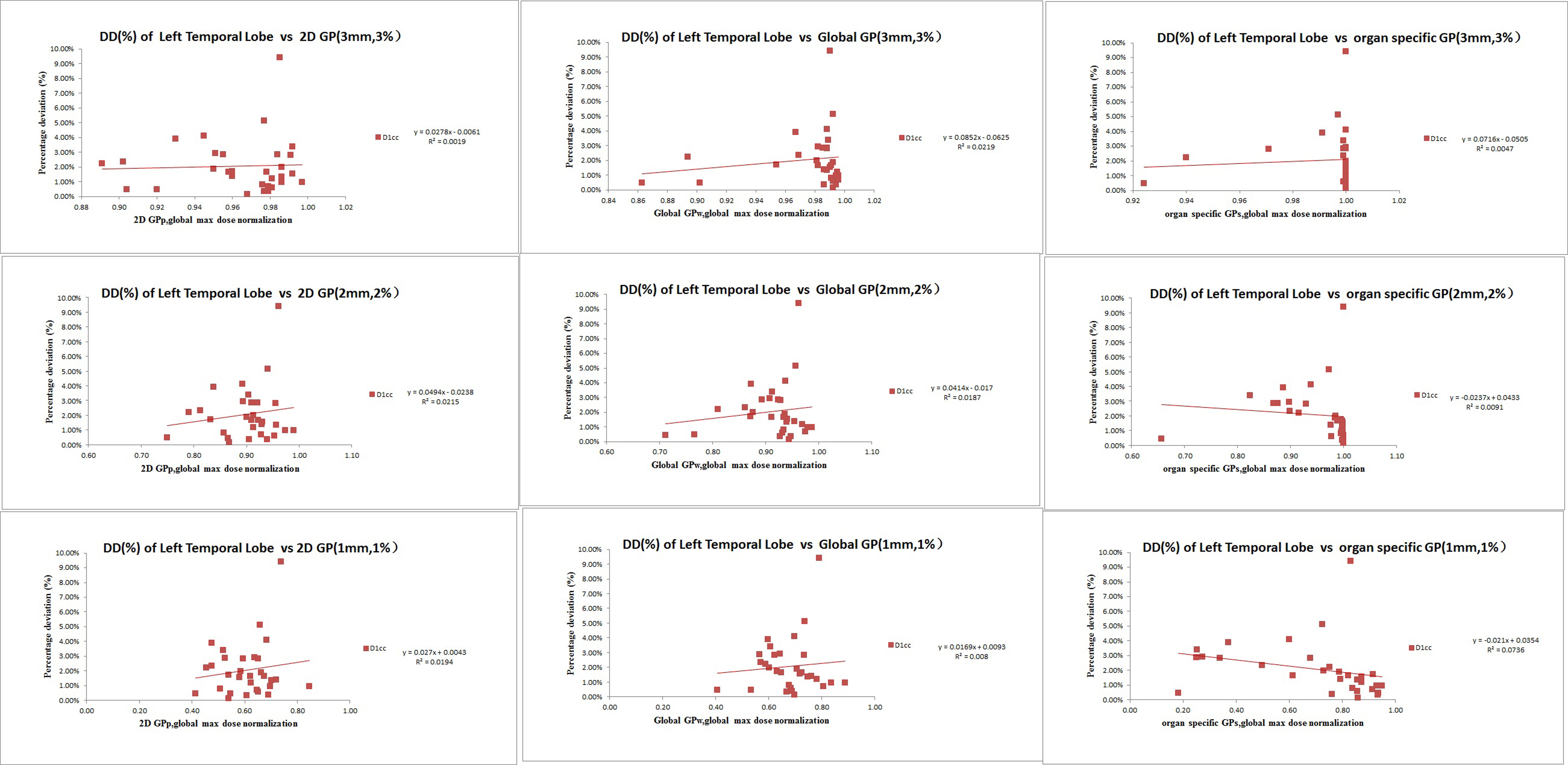

Supplement: Supplementary file 1 — Figure S1. Dose deviation (DD(%)) in PTVnx vs GP (%)-linear fits and R2 were reported. Figure S2. Dose deviation (DD(%)) in PTV1 vs GP (%)-linear fits and R2 were reported. Figure S3. Dose deviation (DD(%)) in PTV2 vs GP (%)-linear fits and R2 were reported. Figure S4. Dose deviation (DD(%)) in spinal cord vs GP (%)-linear fits and R2 were reported. Figure S5. Dose deviation (DD(%)) in Brain stem vs GP (%)-linear fits and R2 were reported. Figure S6. Dose deviation (DD(%)) in left Parotid gland vs GP (%)-linear fits and R2 were reported. Figure S7. Dose deviation (DD(%)) in right Parotid gland vs GP (%)-linear fits and R2 were reported. Figure S8. Dose deviation (DD(%)) in left Temporal lobe vs GP (%)-linear fits and R2 were reported. Figure S9. Dose deviation (DD(%)) in right Temporal lobe vs GP (%)-linear fits and R2 were reported. Figure S10. Dose deviation (DD(%)) in Larynx vs GP (%)-linear fits and R2 were reported. (ZIP 17676 kb) [file 13014_2018_993_MOESM1_ESM.zip › Additional figure-8. Dose deviation in left Temporal lobe vs GP (%)-linear fits and R2 were reported..jpg]

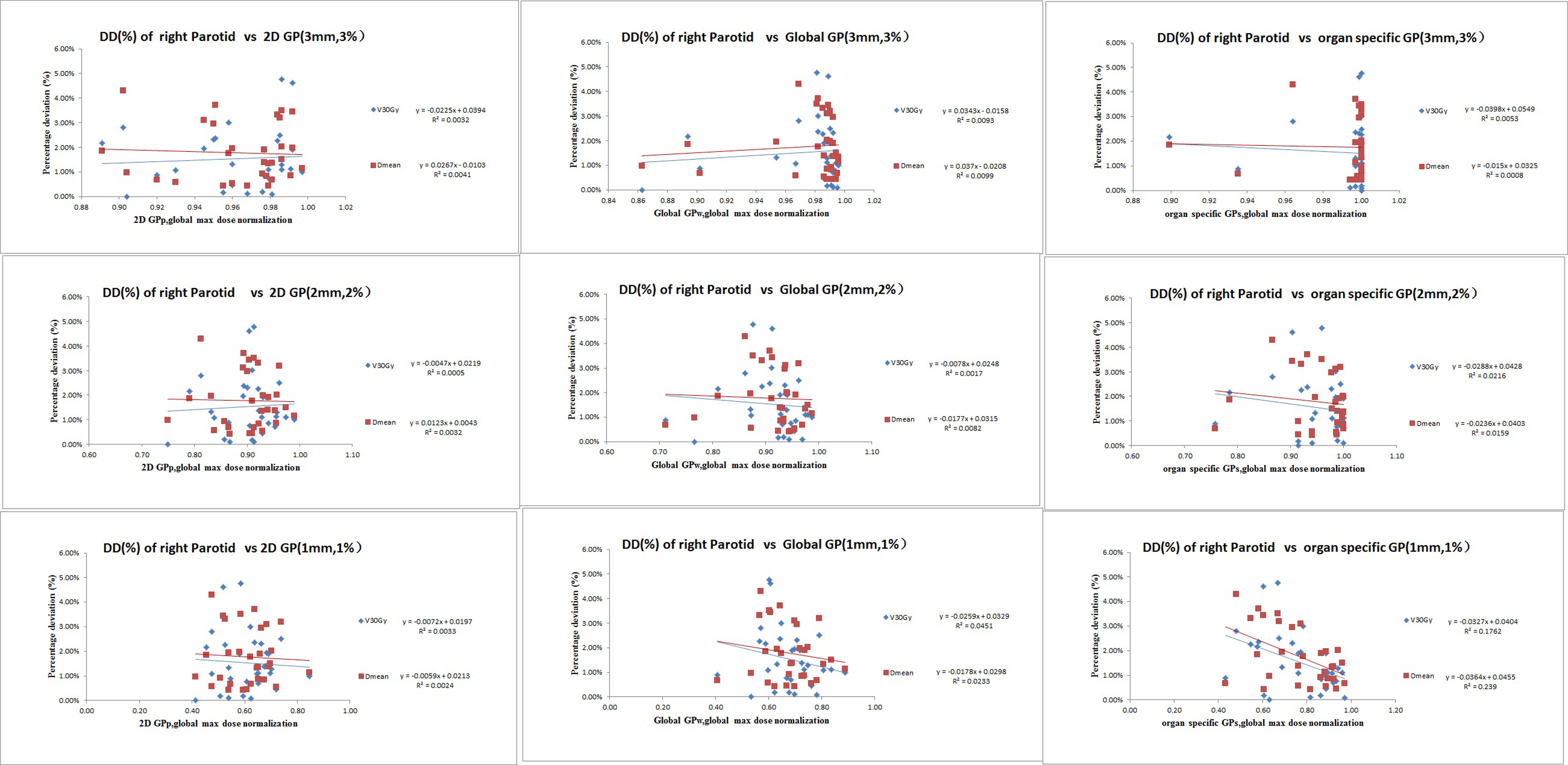

Supplement: Supplementary file 1 — Figure S1. Dose deviation (DD(%)) in PTVnx vs GP (%)-linear fits and R2 were reported. Figure S2. Dose deviation (DD(%)) in PTV1 vs GP (%)-linear fits and R2 were reported. Figure S3. Dose deviation (DD(%)) in PTV2 vs GP (%)-linear fits and R2 were reported. Figure S4. Dose deviation (DD(%)) in spinal cord vs GP (%)-linear fits and R2 were reported. Figure S5. Dose deviation (DD(%)) in Brain stem vs GP (%)-linear fits and R2 were reported. Figure S6. Dose deviation (DD(%)) in left Parotid gland vs GP (%)-linear fits and R2 were reported. Figure S7. Dose deviation (DD(%)) in right Parotid gland vs GP (%)-linear fits and R2 were reported. Figure S8. Dose deviation (DD(%)) in left Temporal lobe vs GP (%)-linear fits and R2 were reported. Figure S9. Dose deviation (DD(%)) in right Temporal lobe vs GP (%)-linear fits and R2 were reported. Figure S10. Dose deviation (DD(%)) in Larynx vs GP (%)-linear fits and R2 were reported. (ZIP 17676 kb) [file 13014_2018_993_MOESM1_ESM.zip › Additional figure-7. Dose deviation in right Parotid gland vs GP (%)-linear fits and R2 were reported..jpg]

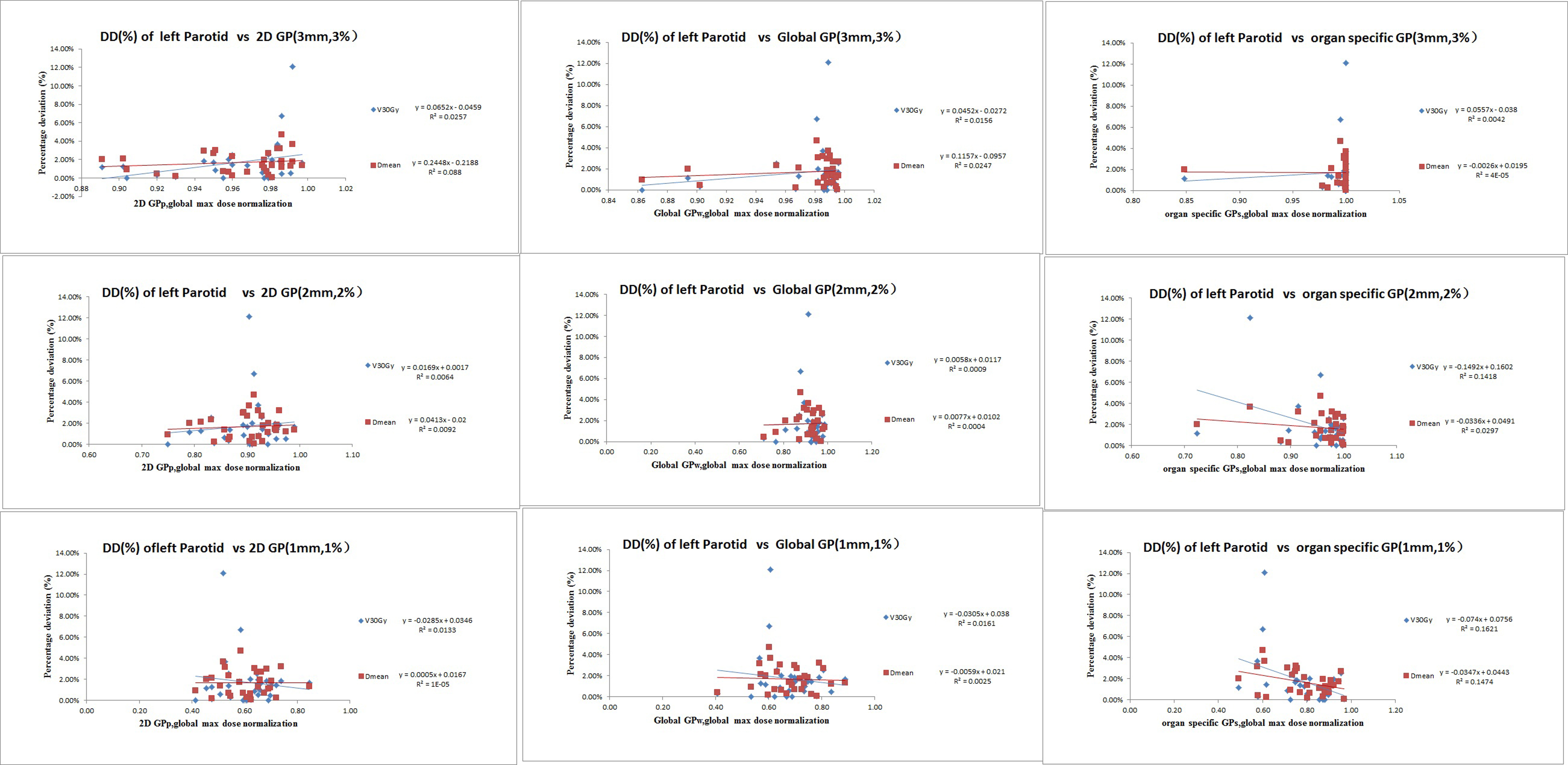

Supplement: Supplementary file 1 — Figure S1. Dose deviation (DD(%)) in PTVnx vs GP (%)-linear fits and R2 were reported. Figure S2. Dose deviation (DD(%)) in PTV1 vs GP (%)-linear fits and R2 were reported. Figure S3. Dose deviation (DD(%)) in PTV2 vs GP (%)-linear fits and R2 were reported. Figure S4. Dose deviation (DD(%)) in spinal cord vs GP (%)-linear fits and R2 were reported. Figure S5. Dose deviation (DD(%)) in Brain stem vs GP (%)-linear fits and R2 were reported. Figure S6. Dose deviation (DD(%)) in left Parotid gland vs GP (%)-linear fits and R2 were reported. Figure S7. Dose deviation (DD(%)) in right Parotid gland vs GP (%)-linear fits and R2 were reported. Figure S8. Dose deviation (DD(%)) in left Temporal lobe vs GP (%)-linear fits and R2 were reported. Figure S9. Dose deviation (DD(%)) in right Temporal lobe vs GP (%)-linear fits and R2 were reported. Figure S10. Dose deviation (DD(%)) in Larynx vs GP (%)-linear fits and R2 were reported. (ZIP 17676 kb) [file 13014_2018_993_MOESM1_ESM.zip › Additional figure-6.Dose deviation in left Parotid gland vs GP (%)-linear fits and R2 were reported..jpg]

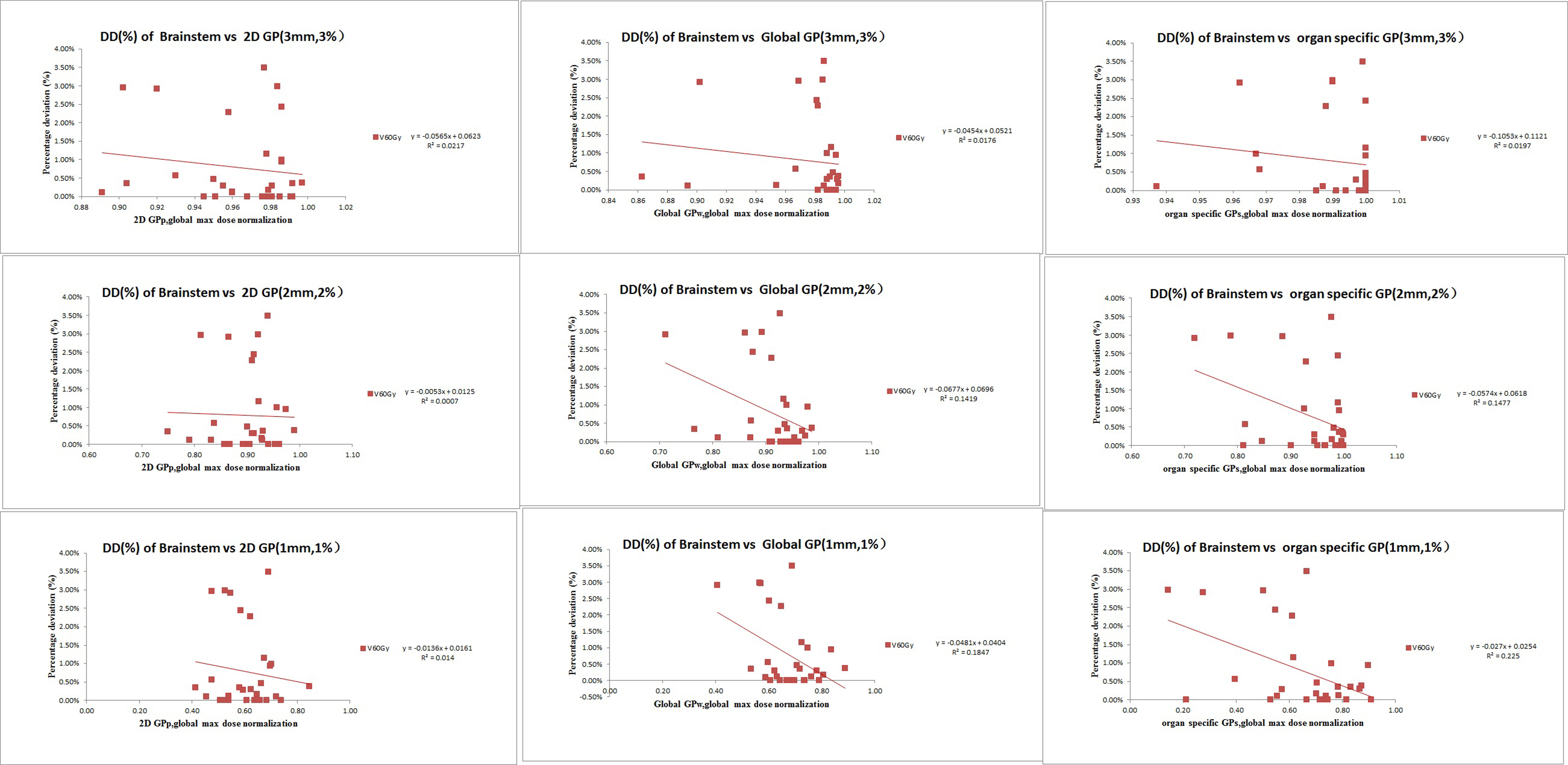

Supplement: Supplementary file 1 — Figure S1. Dose deviation (DD(%)) in PTVnx vs GP (%)-linear fits and R2 were reported. Figure S2. Dose deviation (DD(%)) in PTV1 vs GP (%)-linear fits and R2 were reported. Figure S3. Dose deviation (DD(%)) in PTV2 vs GP (%)-linear fits and R2 were reported. Figure S4. Dose deviation (DD(%)) in spinal cord vs GP (%)-linear fits and R2 were reported. Figure S5. Dose deviation (DD(%)) in Brain stem vs GP (%)-linear fits and R2 were reported. Figure S6. Dose deviation (DD(%)) in left Parotid gland vs GP (%)-linear fits and R2 were reported. Figure S7. Dose deviation (DD(%)) in right Parotid gland vs GP (%)-linear fits and R2 were reported. Figure S8. Dose deviation (DD(%)) in left Temporal lobe vs GP (%)-linear fits and R2 were reported. Figure S9. Dose deviation (DD(%)) in right Temporal lobe vs GP (%)-linear fits and R2 were reported. Figure S10. Dose deviation (DD(%)) in Larynx vs GP (%)-linear fits and R2 were reported. (ZIP 17676 kb) [file 13014_2018_993_MOESM1_ESM.zip › Additional figure-5.Dose deviation in Brain stem vs GP (%)-linear fits and R2 were reported..jpg]

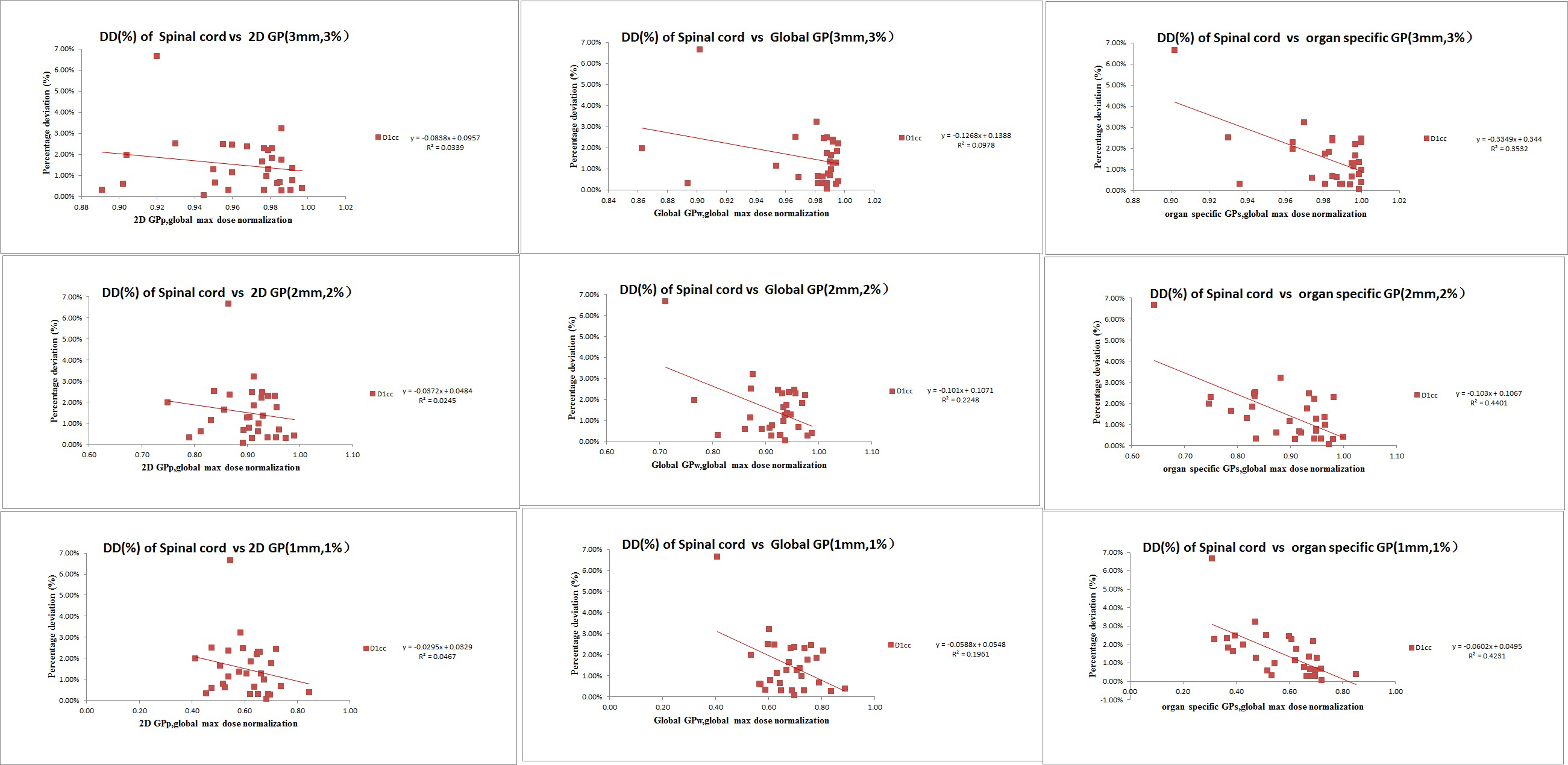

Supplement: Supplementary file 1 — Figure S1. Dose deviation (DD(%)) in PTVnx vs GP (%)-linear fits and R2 were reported. Figure S2. Dose deviation (DD(%)) in PTV1 vs GP (%)-linear fits and R2 were reported. Figure S3. Dose deviation (DD(%)) in PTV2 vs GP (%)-linear fits and R2 were reported. Figure S4. Dose deviation (DD(%)) in spinal cord vs GP (%)-linear fits and R2 were reported. Figure S5. Dose deviation (DD(%)) in Brain stem vs GP (%)-linear fits and R2 were reported. Figure S6. Dose deviation (DD(%)) in left Parotid gland vs GP (%)-linear fits and R2 were reported. Figure S7. Dose deviation (DD(%)) in right Parotid gland vs GP (%)-linear fits and R2 were reported. Figure S8. Dose deviation (DD(%)) in left Temporal lobe vs GP (%)-linear fits and R2 were reported. Figure S9. Dose deviation (DD(%)) in right Temporal lobe vs GP (%)-linear fits and R2 were reported. Figure S10. Dose deviation (DD(%)) in Larynx vs GP (%)-linear fits and R2 were reported. (ZIP 17676 kb) [file 13014_2018_993_MOESM1_ESM.zip › Additional figure-4. Dose deviation in spinal cord vs GP (%)-linear fits and R2 were reported..jpg]

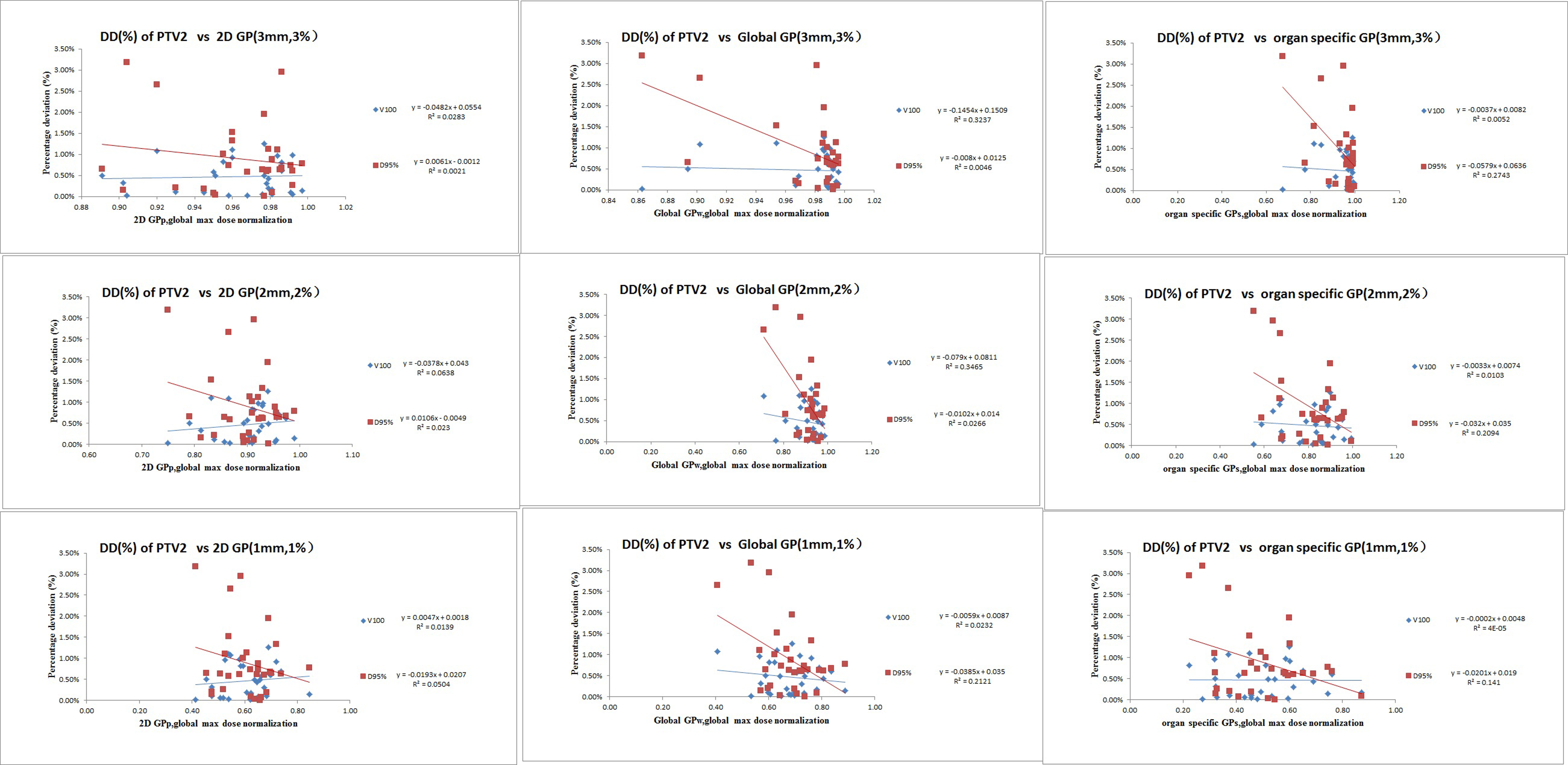

Supplement: Supplementary file 1 — Figure S1. Dose deviation (DD(%)) in PTVnx vs GP (%)-linear fits and R2 were reported. Figure S2. Dose deviation (DD(%)) in PTV1 vs GP (%)-linear fits and R2 were reported. Figure S3. Dose deviation (DD(%)) in PTV2 vs GP (%)-linear fits and R2 were reported. Figure S4. Dose deviation (DD(%)) in spinal cord vs GP (%)-linear fits and R2 were reported. Figure S5. Dose deviation (DD(%)) in Brain stem vs GP (%)-linear fits and R2 were reported. Figure S6. Dose deviation (DD(%)) in left Parotid gland vs GP (%)-linear fits and R2 were reported. Figure S7. Dose deviation (DD(%)) in right Parotid gland vs GP (%)-linear fits and R2 were reported. Figure S8. Dose deviation (DD(%)) in left Temporal lobe vs GP (%)-linear fits and R2 were reported. Figure S9. Dose deviation (DD(%)) in right Temporal lobe vs GP (%)-linear fits and R2 were reported. Figure S10. Dose deviation (DD(%)) in Larynx vs GP (%)-linear fits and R2 were reported. (ZIP 17676 kb) [file 13014_2018_993_MOESM1_ESM.zip › Additional figure-3. Dose deviation in PTV2 vs GP (%)-linear fits and R2 were reported..jpg]

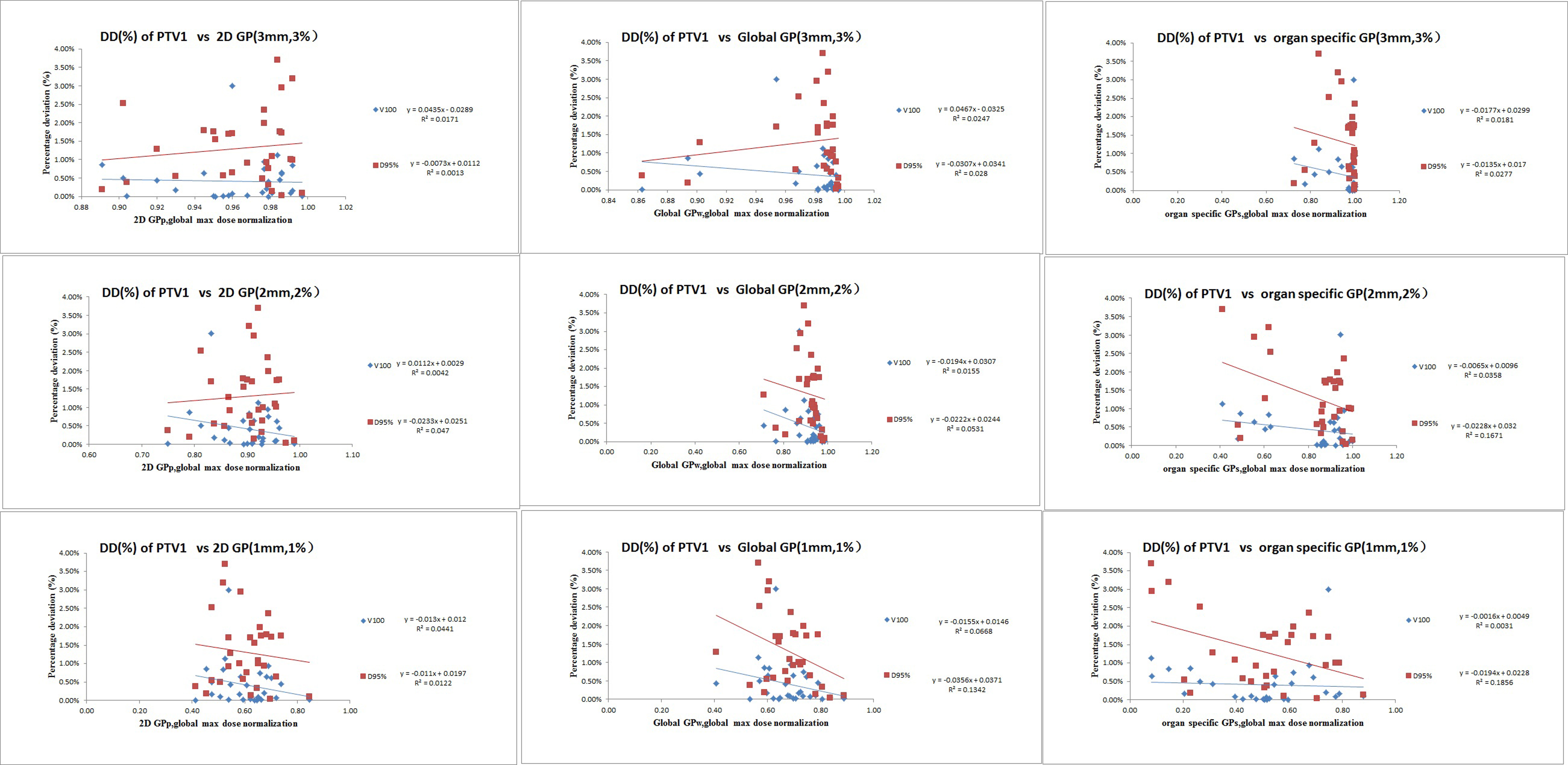

Supplement: Supplementary file 1 — Figure S1. Dose deviation (DD(%)) in PTVnx vs GP (%)-linear fits and R2 were reported. Figure S2. Dose deviation (DD(%)) in PTV1 vs GP (%)-linear fits and R2 were reported. Figure S3. Dose deviation (DD(%)) in PTV2 vs GP (%)-linear fits and R2 were reported. Figure S4. Dose deviation (DD(%)) in spinal cord vs GP (%)-linear fits and R2 were reported. Figure S5. Dose deviation (DD(%)) in Brain stem vs GP (%)-linear fits and R2 were reported. Figure S6. Dose deviation (DD(%)) in left Parotid gland vs GP (%)-linear fits and R2 were reported. Figure S7. Dose deviation (DD(%)) in right Parotid gland vs GP (%)-linear fits and R2 were reported. Figure S8. Dose deviation (DD(%)) in left Temporal lobe vs GP (%)-linear fits and R2 were reported. Figure S9. Dose deviation (DD(%)) in right Temporal lobe vs GP (%)-linear fits and R2 were reported. Figure S10. Dose deviation (DD(%)) in Larynx vs GP (%)-linear fits and R2 were reported. (ZIP 17676 kb) [file 13014_2018_993_MOESM1_ESM.zip › Additional figure-2. Dose deviation in PTV1 vs GP (%)-linear fits and R2 were reported..jpg]

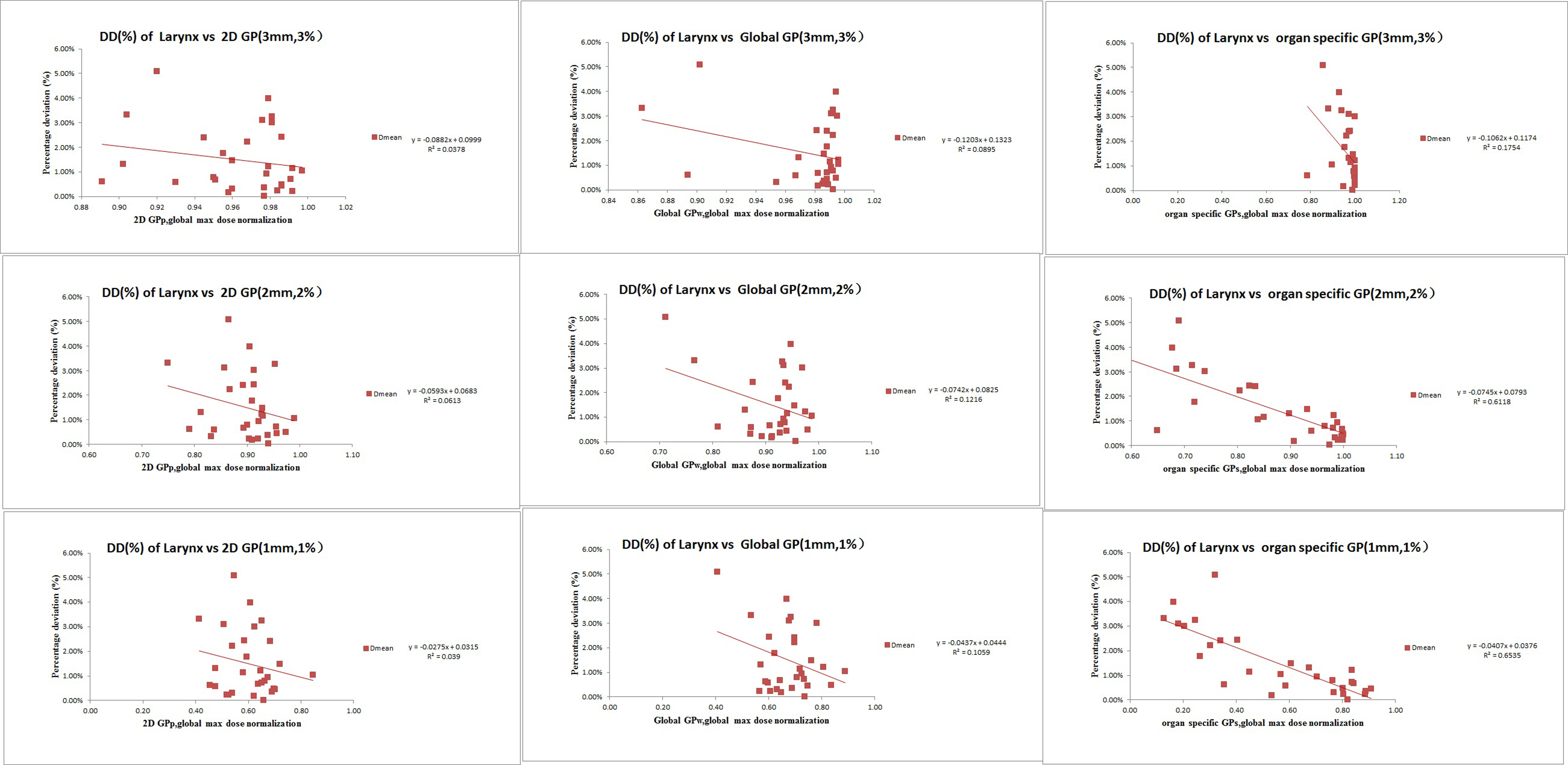

Supplement: Supplementary file 1 — Figure S1. Dose deviation (DD(%)) in PTVnx vs GP (%)-linear fits and R2 were reported. Figure S2. Dose deviation (DD(%)) in PTV1 vs GP (%)-linear fits and R2 were reported. Figure S3. Dose deviation (DD(%)) in PTV2 vs GP (%)-linear fits and R2 were reported. Figure S4. Dose deviation (DD(%)) in spinal cord vs GP (%)-linear fits and R2 were reported. Figure S5. Dose deviation (DD(%)) in Brain stem vs GP (%)-linear fits and R2 were reported. Figure S6. Dose deviation (DD(%)) in left Parotid gland vs GP (%)-linear fits and R2 were reported. Figure S7. Dose deviation (DD(%)) in right Parotid gland vs GP (%)-linear fits and R2 were reported. Figure S8. Dose deviation (DD(%)) in left Temporal lobe vs GP (%)-linear fits and R2 were reported. Figure S9. Dose deviation (DD(%)) in right Temporal lobe vs GP (%)-linear fits and R2 were reported. Figure S10. Dose deviation (DD(%)) in Larynx vs GP (%)-linear fits and R2 were reported. (ZIP 17676 kb) [file 13014_2018_993_MOESM1_ESM.zip › Additional figure-10.Dose deviation in Larynx vs GP (%)-linear fits and R2 were reported..jpg]

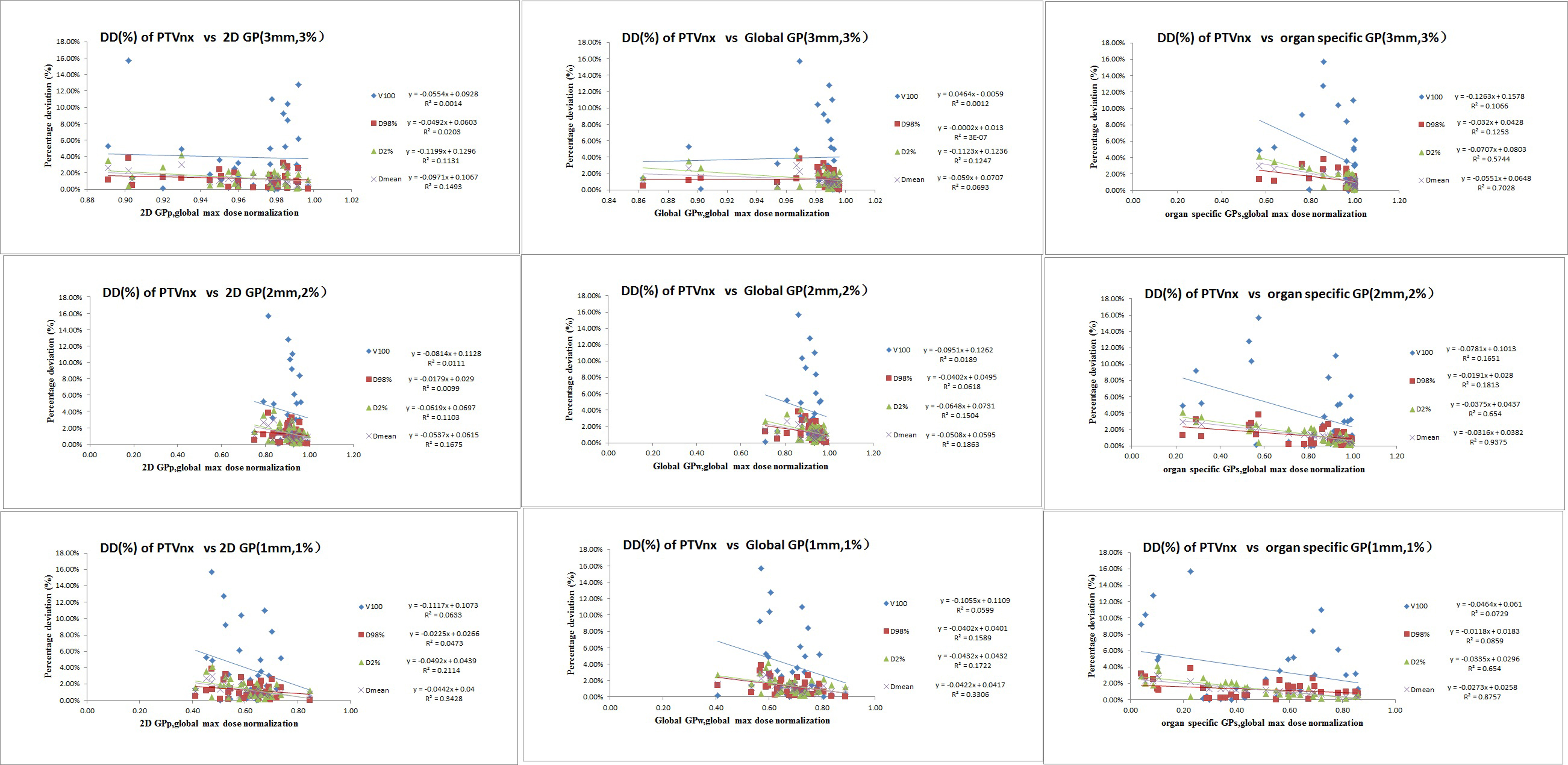

Supplement: Supplementary file 1 — Figure S1. Dose deviation (DD(%)) in PTVnx vs GP (%)-linear fits and R2 were reported. Figure S2. Dose deviation (DD(%)) in PTV1 vs GP (%)-linear fits and R2 were reported. Figure S3. Dose deviation (DD(%)) in PTV2 vs GP (%)-linear fits and R2 were reported. Figure S4. Dose deviation (DD(%)) in spinal cord vs GP (%)-linear fits and R2 were reported. Figure S5. Dose deviation (DD(%)) in Brain stem vs GP (%)-linear fits and R2 were reported. Figure S6. Dose deviation (DD(%)) in left Parotid gland vs GP (%)-linear fits and R2 were reported. Figure S7. Dose deviation (DD(%)) in right Parotid gland vs GP (%)-linear fits and R2 were reported. Figure S8. Dose deviation (DD(%)) in left Temporal lobe vs GP (%)-linear fits and R2 were reported. Figure S9. Dose deviation (DD(%)) in right Temporal lobe vs GP (%)-linear fits and R2 were reported. Figure S10. Dose deviation (DD(%)) in Larynx vs GP (%)-linear fits and R2 were reported. (ZIP 17676 kb) [file 13014_2018_993_MOESM1_ESM.zip › Additional figure-1. Dose deviation in PTVnx vs GP (%)-linear fits and R2 were reported..jpg]
