# Supplementary material for: Retrospective dosimetry study of intensity-modulated radiation therapy for nasopharyngeal carcinoma: measurement-guided dose reconstruction and analysis
Source: Radiat Oncol. 2018 Mar 15;13:42. doi: 10.1186/s13014-018-0993-2 (PMC5856312; doi:10.1186/s13014-018-0993-2)
Supplement: Supplementary file 2 — Table S1. The comparison of the 3D globe, and organ-specific GP (%) calculated with local dose normalization for 30 NPC cases with different gamma criteria. Table S2. Pearson correlation coefficient with three type gamma pass rate calculated with local dose normalization and DV, VD. Table S3. Significant p-values for correlation between three type gamma pass rate and DV, VD. (ZIP 55 kb) [file 13014_2018_993_MOESM2_ESM.zip › Additional table 2.docx]

Additional table 2 Pearson correlation coefficient with three type gamma pass rate calculated with local dose normalization and D_V_, V_D_.

|  | DVH | global 3D GP | | | | organ specific 3D GP | | | | | |
| --- | --- | --- | --- | --- | --- | --- | --- | --- | --- | --- | --- |
| structure | index | 3mm/3% | | 2mm/2% | 1mm/1% | 3mm/3% | | 2mm/2% | | 1mm/1% | |
| PTVnx | V100% | -0.18 | -0.266 | | -0.276 | | -0.37 | | -0.412 | | -0.079 |
|  | D98% | -0.226 | -0.303 | | -0.332 | | -0.394 | | -0.433 | | -0.178 |
|  | D2% | -0.386 | -0.379 | | -0.368 | | -0.751 | | **-0.804*** | | -0.79 |
|  | Dmean | -0.482 | -0.518 | | -0.518 | | **-0.865*** | | **-0.976*** | | **-0.827*** |
| PTV1 | V100% | -0.155 | -0.15 | | -0.276 | | -0.183 | | -0.179 | | -0.057 |
|  | D95% | -0.151 | -0.266 | | -0.361 | | -0.179 | | -0.408 | | -0.424 |
| PTV2 | V100% | -0.006 | -0.082 | | -0.118 | | -0.132 | | -0.053 | | -0.002 |
|  | D95% | -0.386 | -0.329 | | -0.253 | | -0.474 | | -0.409 | | -0.357 |
| SC | D1cc | -0.355 | -0.41 | | -0.366 | | -0.417 | | -0.57 | | -0.612 |
| BS | V60 Gy | -0.227 | -0.272 | | -0.207 | | 0.016 | | -0.298 | | -0.429 |
| Parotid-L | Dmean | 0.058 | 0.036 | | -0.032 | | -0.012 | | -0.059 | | -0.389 |
|  | V30 Gy | 0.046 | -0.044 | | -0.136 | | 0.074 | | -0.023 | | -0.391 |
| Parotid-R | Dmean | -0.026 | -0.059 | | -0.094 | | -0.084 | | -0.148 | | -0.517 |
|  | V30 Gy | 0.092 | 0 | | -0.054 | | -0.088 | | -0.076 | | -0.418 |
| TL-L | D1cc | -0.062 | -0.036 | | -0.091 | | -0.568 | | -0.605 | | -0.608 |
| TL-R | D1cc | -0.143 | -0.114 | | -0.12 | | -0.649 | | -0.683 | | -0.609 |
| Larynx | Dmean | -0.381 | -0.378 | | -0.288 | | -0.635 | | -0.783 | | **-0.807*** |

The two-tailed Student t-test with SPSS statistical software (V19);

**Bold** letters in the data highlighted R>0.8 and * symbolled the significance of the correlation at the 0.01 level (*P* <0.01, bilateral)

**D_V_**- volumetric dose, **V_D_** - dose volumes; **PTV** - planning target volume, **GP** (%) – gamma pass rate (%), **SC**-spinal cord, **BS** – brainstem, **Parotid-L** – left parotid gland, **Parotid-R** – right parotid gland, **TL-L**-left temporal lobe, **TL-R** - right temporal lobe.
